# Characterization of antiviral T cell responses during primary and secondary challenge of laboratory cats with feline infectious peritonitis virus (FIPV)

**DOI:** 10.1186/s12917-019-1909-6

**Published:** 2019-05-22

**Authors:** Farina Mustaffa-Kamal, Hongwei Liu, Niels C. Pedersen, Ellen E. Sparger

**Affiliations:** 1 Department of Medicine and Epidemiology, University of California, One Shields Avenue, Davis, CA 95616 USA; 2 Department of Veterinary Pathology and Microbiology, Faculty of Veterinary Medicine, Universiti Putra Malaysia, 43400 Serdang, Selangor Malaysia; 3 Center for Companion Animal Health, University of California, One Shields Avenue, Davis, CA 95616 USA

**Keywords:** Feline infectious peritonitis, Feline infectious peritonitis virus, Antiviral T cell responses

## Abstract

**Background:**

Feline infectious peritonitis (FIP) is considered highly fatal in its naturally occurring form, although up to 36% of cats resist disease after experimental infection, suggesting that cats in nature may also resist development of FIP in the face of infection with FIP virus (FIPV). Previous experimental FIPV infection studies suggested a role for cell-mediated immunity in resistance to development of FIP. This experimental FIPV infection study in specific pathogen free (SPF) kittens describes longitudinal antiviral T cell responses and clinical outcomes ranging from rapid progression, slow progression, and resistance to disease.

**Results:**

Differences in disease outcome provided an opportunity to investigate the role of T cell immunity to FIP determined by T cell subset proliferation after stimulation with different viral antigens. Reduced total white blood cell (WBC), lymphocyte and T cell counts in blood were observed during primary acute infection for all experimental groups including cats that survived without clinical FIP. Antiviral T cell responses during early primary infection were also similar between cats that developed FIP and cats remaining healthy. Recovery of antiviral T cell responses during the later phase of acute infection was observed in a subset of cats that survived longer or resisted disease compared to cats showing rapid disease progression. More robust T cell responses at terminal time points were observed in lymph nodes compared to blood in cats that developed FIP. Cats that survived primary infection were challenged a second time to pathogenic FIPV and tested for antiviral T cell responses over a four week period. Nine of ten rechallenged cats did not develop FIP or T cell depletion and all cats demonstrated antiviral T cell responses at multiple time points after rechallenge.

**Conclusions:**

In summary, definitive adaptive T cell responses predictive of disease outcome were not detected during the early phase of primary FIPV infection. However emergence of antiviral T cell responses after a second exposure to FIPV, implicated cellular immunity in the control of FIPV infection and disease progression. Virus host interactions during very early stages of FIPV infection warrant further investigation to elucidate host resistance to FIP.

## Background

Naturally occurring feline infectious peritonitis (FIP) is invariably fatal once clinical signs appear [1]. However, mortality due to feline infectious peritonitis virus (FIPV) infection may not be nearly as severe as reports in the field indicate, because many infected cats may not show overt clinical disease and therefore escape diagnosis. The proportion of naturally infected cats that are resistant to disease is difficult to estimate, although it is probably similar to the proportion of random bred cats that fail to develop FIP after experimental infection. Depending on the strain of FIPV and the route of inoculation used for experimental infection, up to 36% of random bred cats will resist disease [2]. The nature of this immunity is unknown, although humoral immunity is known to be non-protective and contributory to the disease signs [3]. Multiple studies have led researchers to conclude that immunity to FIPV is largely cell-mediated [4–8]. The potential role of cellular immunity in FIP was also inferred from experiments with cats rendered immunocompromised by chronic experimentally-induced feline immunodeficiency virus (FIV) infection. Twenty percent of FIV-infected cats developed FIP upon infection with feline enteric coronavirus (FECV), the parental virus to FIPV, whereas none of the FIV naive siblings became ill [9]. This study suggested that although the FECV to FIPV mutation is quite common in nature, FIP is unlikely to develop in older cats in the face of a normal T-cell immunity.

The first study reporting virus-specific immune responses in cats with recurrent FIPV infection revealed that multiple epitopes within the spike (S) protein encoded by FIPV, were targeted by both CD4 and CD8 T cells [6]. However, the predominant response was observed in CD8 T cells, particularly in cats that survived a type II FIPV-79-1146 infection. Later reports based on experimental infection with type II FIPV-79-1146 and type I FIPV-KU2 inoculation demonstrated that expression of interferon-gamma (IFN-γ) by antiviral T cells was associated with resistance to the development of FIP [7, 8]. These studies also identified multiple epitopes encoded within the S-2 domain of the viral spike protein and the N protein that were associated with Th1 responses and resistance to the development of FIP. In spite of these and other studies, the body of evidence suggesting the importance of virus-specific T cell responses in resistance to FIPV-associated disease remains small, especially for pathogenic type I FIPV isolates. Given that type I FIPV is the most common feline coronavirus in the field, additional studies characterizing virus-specific cellular immune responses during type I FIPV infection seem particularly warranted [10–14] .

This report describes the characterization of antiviral T cell responses of multiple cohorts of cats involved in experimental FIPV infection studies. One group of cats developed FIP following experimental primary FIPV infection with disease distinguished by two different outcomes including rapid or slow progression. A small proportion of cats within the same experimental infection study, proved resistant to disease demonstrating a third possible outcome during primary experimental FIPV infection. A cohort of cats resistant to development of FIP during primary infection were re-inoculated with FIPV to examine resistance and cellular immune responses associated with a secondary virus challenge. Antiviral T cell responses observed for these different infection outcomes were compared to characterize possible virus-specific T cell immune correlates for resistance to the development of FIP. Although a unique antiviral T cell response during early acute infection could not be correlated with resistance or susceptibility to FIP, differences in antiviral T cell responses were observed for primary versus rechallenge exposures to FIPV.

## Methods

### Ethics statement

Specific pathogen-free (SPF) cats were obtained from the breeding colony of the Feline Nutrition and Pet Care Center, School of Veterinary Medicine, University of California, Davis, CA. Animals were housed and maintained according to regulations and guidelines of the University of California Davis Institutional Animal Care and Use Committee (UC Davis IACUC approval numbers 15,309 and 16,637).

### Cats and FIPV infection

Virus naive SPF cats (*n* = 19) aged five to six months and designated as a primary infection group were inoculated by the oro-nasal route with FIPV-i3c2, a tissue-derived isolate from experimental cats infected with field virus as previously described [15]. Virus inoculum contained semi-purified cell-free supernatants of finely ground diseased tissues including omentum from FIPV-i3c2-infected cats. Each cat received an inoculum containing 1 ml of a 1:5–1:10 dilution of a 25% cell-free suspension which has proved infectious to 100% of cats based on occurrence of disease and/or seroconversion. Ten FIPV-resistant cats aged 8–24 months derived from previous experimental infection studies and designated as survivors from confirmed primary infections with infectious FIPV-i3c2, were rechallenged using similar FIPV inocula and infection route to establish a rechallenge infection group. The amount of time elapsed between primary FIPV infection and rechallenge varied for the survivor cat cohort. One subgroup of the survivor cohort involved six cats that received a primary inoculation of FIPV-i3c2 by the oro-nasal route two to six months prior to rechallenge. Four of these six cats in this subgroup included survivors derived from the primary infection group described within this report. A second subgroup included four cats that received 1–3 inoculations of FIPV-i3c2 delivered by either oro-nasal or intraperitoneal routes more than 12 months prior to rechallenge. Twelve uninfected healthy SPF cats aged five to six months served as controls for these studies. Each cat was followed from pre-bleed (week 0) until week 4 post-infection (PI) or the time of euthanasia due to the development of FIP, with weekly blood samples collected for complete blood count (CBC), T cell counts, and virus-specific T cell immune response assays. Inoculated cats were also monitored daily for rectal temperature, fever, inappetance, depression, diarrhea, dehydration, ascites, hyperbilirubinuria and jaundice. Cats showing symptoms of FIP were euthanatized with an intravenous overdose of pentabarbital/phenytoin. Mesenteric lymph nodes (MLN), peripheral lymph nodes (PLN) and blood for peripheral blood mononuclear cells (PBMC) and plasma isolation were collected in cats undergoing euthanasia due to onset of FIP and from two healthy uninfected control cats. These experimental cat cohorts were part of a larger experimental infection study testing FIPV pathogenesis according to different FIPV variants and prior infection with either FIPV or FECV [2]. Cats were maintained in housing free of other feline pathogens and cared for in a uniform manner.

### Isolation of PBMC and mononuclear cells from lymph nodes

PBMC were isolated from blood samples collected from infected cats at weekly time points by density centrifugation using Ficoll-Histopaque (Sigma-Aldrich, St. Louis, MO). Mononuclear cells were isolated from lymph node tissues (LNMC) harvested post mortem from cats that developed FIP as previously described [16]. PBMC and LNMC were incubated overnight in PBMC media [17] without interleukin (IL)-2 and assayed for T cell phenotype and FIPV-specific T cell responses the following day.

### T-cell phenotypic analysis

CD3+ lymphocyte (T cell) counts were based on T cell frequencies within PBMC preparations determined by multicolor flow cytometry and total lymphocyte counts derived from CBCs. PBMC were assayed for T cell frequency by staining with either a biotinylated or Alexa Fluor (AF)647-conjugated anti-human CD3 monoclonal antibody (CD3–12) (AbD Serotec, Raleigh, NC) specific for a conserved intracellular epitope shown to be cross-reactive for multiple species including feline CD3 [18, 19] and analysis by flow cytometry. Biotinylated CD3 antibody-staining cells were detected with a strepavidin-phycoerythrin (PE)-Cy7 conjugate (Invitrogen, Valencia, CA). Viability of cells acquired for flow cytometric analysis was assessed using the Live/Dead® Fixable Aqua stain (Invitrogen). Data were acquired using an LSRII flow cytometer (BD Biosciences, San Diego, CA) with 50,000 events obtained for each sample and analyzed using FlowJo software (TreeStar Inc., Ashland, OR).

### Indirect immunofluorescent antibody staining (IFA)

Serum samples collected from FIPV-inoculated cats and uninfected controls were tested for viral antibody by an indirect immunofluorescence antibody (IFA) assay as previously described [20] with commercial slides coated with FIPV type II-infected cells (VMRD, Pullman, WA). This assay was performed according to manufacturer’s recommendation at dilutions of 1:25, 1:100, 1:400, 1:1600 and 1:6400. Slides were assayed using fluorescence microscopy (Olympus BX51, Olympus, Center Valley, PA) and titers were reported based on the highest dilution up to 1:6400 that yielded detectable fluorescence in foci of infected cells.

### Virus detection

Two different extraction methods were used for nucleic acid extraction from cells and tissues in this study. One method for viral nucleic acid extraction from PBMC used RLT lysis buffer (Qiagen, Valencia, CA) (with beta mercaptoethanol as per manufacturer’s instructions). The resulting lysate was protein-digested at 56 °C for 30 min and extracted for RNA and DNA utilizing a commercial kit (Vet-For-All; Qiagen) and a BioSprint 96 magnetic bead extraction (Qiagen) according to the manufacturer’s protocol. Viral RNA extraction from plasma (140 μl) also utilized RLT lysis buffer, but RNA was extracted using the QIAamp Viral RNA Mini kit (Qiagen). LNMC from mesenteric lymph nodes were lysed in AB lysis buffer (Applied Biosystems, Grand Island, NY) and RNA extraction was performed using the ABI Prism 6100 (Life Technologies, Grand Island, NY) based on protocols previously described [21]. The protocol for cDNA synthesis was previously described [22]. FIPV viral loads were assayed in plasma, PBMC, and LNMC (mesenteric lymph node) by TaqMan real-time qPCR using a primer probe set based on the conserved feline coronavirus 7b gene with a minor modification of the published probe set sequence [23] as follows: AGAGAAGTTTAAAGATCCGC. TaqMan real-time qPCR was performed using the 7900 HTFast System AB TaqMan real-time qPCR. Viral RNA loads in plasma were derived from a standard curve established with a plasmid (pCR 2.1, Operon) containing the 7b gene target sequence. Virus loads in PBMC were expressed as viral RNA copies per million PBMC determined by normalization of viral copies against feline chemokine receptor (CCR)5 copy number measured for DNA extracted from the same cell preparation [24]. Based on standard curves established with the feline coronavirus 7b and feline CCR5 plasmids, the detection limit for either viral RNA or CCR5 DNA was 10 copies per reaction. Viral RNA loads in LNMC were calculated by the 2-ΔΔCt method [25] normalizing viral RNA expression against feline glyceraldehyde 3-phosphate dehydrogenase (GAPDH) expression measured for each LNMC RNA sample.

### Viral antigens for T cell immune response assays

A panel of 54 peptides derived from the heptad region (HR) 1 and inter-helical (IH) region of spike 2 (S2) domain of the type I FIPV-UCD11 spike protein (accession number: FJ917519) [26] were used as viral antigens in a virus-specific T cell proliferation assay (Table 1). Peptides (Sigma-Aldrich) were synthesized as 15-mer fragments with an overlap of 11 amino acids. The lyophilized peptides were dissolved in 10% dimethyl sulfoxide (DMSO), pooled at an approximate concentration of 21.6 mg/ml and stored at − 80 °C. For generation of whole-killed virus (WKV) antigen, FIPV-UCD 1 [5] virus stocks were prepared from infected *Felis catus* whole fetus-4 (fcwf-4) cell (ATCC) cultures. Virus was precipitated from culture supernatants using polyethylene glycol (PEG) and high speed centrifugation, and inactivated by ultraviolet (UV) irradiation for 15 min. Western blot and infectivity assays using fcwf-4 cells were performed to confirm the presence of virus particles and virus inactivation for WKV preparations respectively.

**Table 1:** Amino acid sequences of peptides derived from type 1 FIPV spike protein

Amino acid sequence (start position)	
HR1 peptides	IH peptides
TSAVAVPFAMQVQARLNY (1055)	ITGRLAALNAYVSQTLTQYA (1175)
VALQTDVLQENQKILANA (1073)	EVKASRQLAMEKVNECVKS (1195)
FNNAIGNITLALGKVSNSI (1091)	QSDRYGFCGNGTHLFSLVN (1214)
TTISDGFNTMASALTKIQS (1110)	SAPEGLLFFHTVLLPTEWEE (1233)
VVNQQGEALSQLTSQLQ (1129)	VTAWSGICVNNTYAYVLKDF (1253)
KNFQAISSSIAEIYNRLEK (1146)	EHSIFSYNNTY (1273)
VEADAQVDRL (1165)	

### Virus-specific T cell proliferation assay

Virus-specific T cell proliferation responses in PBMC and LNMC isolated from FIPV-inoculated cats and uninfected controls were assessed using a commercial kit based on bromodeoxyuridine (BrdU) incorporation (BrdU Flow Kit; BD Biosciences) and a previously described protocol [27] with modifications. Briefly, freshly isolated PBMC and LNMC were plated at 10^6^ cells per well in a 24 well plate in standard PBMC media without IL-2 and cultured (rested) overnight at 37 °C. The next day cells were stimulated with 2 μg WKV or an overlapping peptide pool derived from spike HR1 and IH regions with a working concentration of 2 μg/ml. Unstimulated cells cultured in PBMC media without IL-2 served as a negative control. Cells were incubated with 10 μM BrdU for 24 h prior to harvesting and then stained for BrdU incorporation using a commercial anti-BrdU antibody labeled with allophycocyanin (APC) (BD Biosciences) according to manufacturer’s instructions. For cell surface markers, cells were stained with anti-feline CD4 (clone 34F4) (Southern Biotech, Birmingham, AL) conjugated to Pacific Blue using a commercial kit (Molecular Probes, Grand Island, NY) and anti-feline CD8- PE (clone vpg9) (AbD Serotec) and assessed for viability with Live/Dead® Fixable Aqua stain (Invitrogen). Cells were fixed and permeabilized according to manufacturer’s instructions and next stained for intracellular CD3 expression using anti-human CD3- fluorescein isothiocyanate (FITC) (clone CD3–12) (AbD Serotec) and BrdU incorporation with an APC-labeled anti-BrdU antibody (BD Biosciences). Data were acquired using an LSRII flow cytometer (BD Biosciences) and 100,000 events were collected and analyzed for each sample using FlowJo software (TreeStar, Inc). Frequencies for viral immunogen-stimulated proliferation were adjusted by subtraction of frequencies measured for unstimulated control PBMC preparations for each sample to yield a final value for frequencies of antigen-specific T cells.

### Statistical analysis

Statistical analysis comparing trends for differential blood and T cell counts determined for cats during primary and rechallenge infection over time was based on linear mixed effects regression models using random effects for subject to account for repeated measures (R, 2.11.0). A Kruskal Wallis test was used to compare lymphocyte and T cell counts between three different disease outcomes during primary FIPV infection (GraphPad Software, Inc., San Diego CA). Pair-wise analysis was performed to compare median frequencies of proliferating CD4 and CD8 T cells after stimulation with viral immunogens using the Mann-Whitney U test (one-tailed) also using GraphPad Prism. *P* values < 0.05 were considered significant.

## Results

### Disease outcome

Nineteen naive SPF cats were inoculated oronasally with the FIPV-i3c2 isolate and monitored for illness up to 106 days post-infection. Fifteen cats (79%) succumbed to FIP during primary infection while the remaining four cats (21%) were still healthy without fever or clinical signs of FIP until the end of the study (106 days PI) and designated FIP resistant or survivors. The median survival for those cats that developed FIP during primary FIPV-i3c2 infection was 43.5 days. Eleven of the 15 diseased cats (73%) manifested the effusive form (wet) of FIP characterized by ascites and inflammation of intestinal serosa and 4/15 (27%) developed the non-effusive (dry or wet-dry) form characterized by granulomatous lesions in abdominal organs, central nervous system, or both tissues. Eight of 11 cats with effusive FIP died within 30 days and were deemed rapid progressors (Table 2). Three cats with effusive FIP and the four cats with non-effusive FIP survived past 30 days and were designated slow progressors (Table 2). Overall, 8/19 (42%) of the experimentally infected cats were classified as rapid progressors, 7/19 (37%) slow progressors, and 4/19 (21%) as FIP resistant (survivors). Ten cats that survived primary infection with FIPV-i3c2, including four survivor cats from this acute infection study, were challenged again with the same FIPV isolate. One out of the ten (10%) cats succumbed to FIP within three weeks of rechallenge (Table 3). Importantly, the remaining nine cats within the rechallenge group did not develop FIP based on the absence of FIP-associated symptoms after a secondary exposure to virus.

**Table 2:** Summary of findings for primary FIPV infection

Cat ID	Onset of fever (dpi)	Time of euthanasia (dpi)	FIP form	Antibody titer	Progressor type	Virus load
11–163	2	9	Wet	1:25	Rapid	BLD
11–165	7	11	Wet	1:100	Rapid	BLD
11–224	13	15	Wet	1:100	Rapid	6.1 × 10^2^ PB W1
11–226	13	15	Wet	1:25	Rapid	BLD
11–227	15	24	Wet	1:100	Rapid	4.8 × 10^4^ P W2
11–150	28	29	Wet	1:400	Rapid	BLD
11–162	28	29	Wet	1:400	Rapid	8.7 × 10^4^ P W2
11–230	28	30	Wet	< 1:25	Rapid	4.1 × 10^4^ P TB
11–152	25	42	Wet-dry	1:400	Slow	4.3 × 10^2^ PB W2 3.5 × 10^2^ PB W3
11–228	40	45	Dry	1:100	Slow	BLD
11–229	39	45	Wet-dry	1:400	Slow	BLD
11–231	46	48	Wet	1:100	Slow	BLD
11–164	35	49	Wet	1:1600	Slow	1.5 × 10^6^ P W2 6.1 × 10^3^ PB W2
11–151	39	50	Wet	1:400	Slow	BLD
11–148	28	106	Dry	1:6400	Slow	2.5 × 10^4^ PB W2 3.9 × 10^2^ PB W3
11–147	NA	NA	NA	1:100	FIP resistant	3.1 × 10^4^ P W2
11–149	NA	NA	NA	1:100	FIP resistant	3.2 × 10^5^ P W2 1.8 × 10^2^ PB W2
11–166	NA	NA	NA	1:100	FIP resistant	BLD
11–225	NA	NA	NA	1:400	FIP resistant	BLD

The abbreviation dpi represents days post inoculation. Antibody titers represent the time point of euthanasia or 4 weeks post inoculation for survivor cats. Virus load represents measurements in PBMC and plasma only. BLD denotes below the limits of detection. PB represent PBMC and the associated value shown represents FIPV RNA copies per 10^6^ cells. P denotes plasma and the associated value represents FIPV RNA copies per ml. W with associated number value stands for week PI and TB stands for terminal bleed. Virus load detection results in LNMC are reported in the text

**Table 3:** FCoV antibody titer after FIPV rechallenge

Cat ID	Antibody titer after primary infection	Antibody titer after rechallenge infection
10–068	1:25	1:100
10–084	1:100	1:400
10-143^a^	1:25	1:100
10–145	1:25	1:100
11–073	1:400	1:1600
11–074	1:400	1:1600
11–147	1:1600	1:6400
11–149	1:400	1:1600
11–166	1:1600	1:6400
11–225	1:1600	1:6400

^a^Cat died as a result of FIP at week 3 post FIPV rechallenge. For all other cats, antibody titers after rechallenge represent 4 weeks after re-inoculation with virus whereas titers after primary infection represent day 0 of the rechallenge study

### Differential cell counts and T cell measurements

**Fig. 1:**
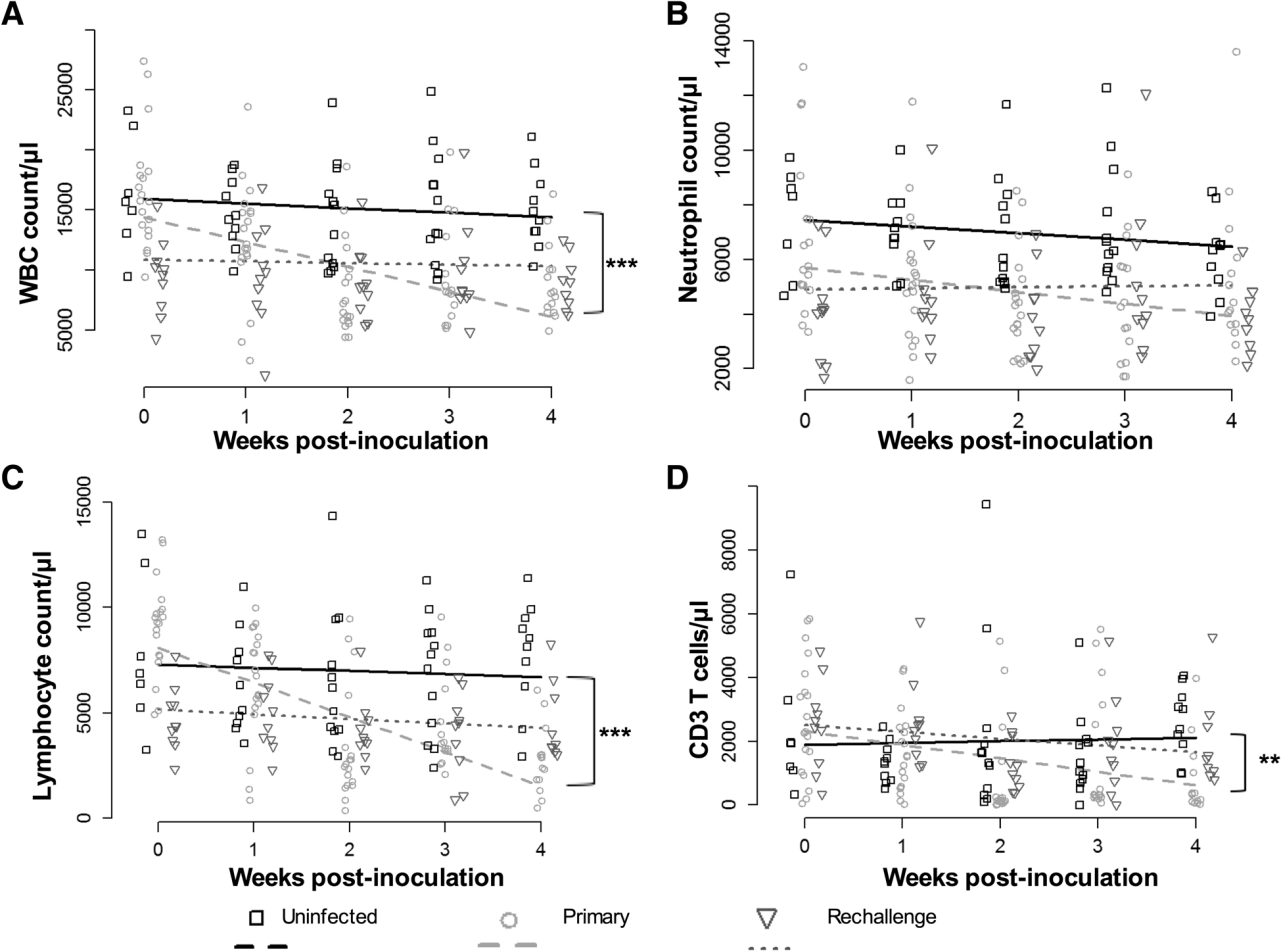
Comparison of differential cell counts including T cell counts in different FIPV infection cohorts. A linear mixed regression analysis of differential blood leukocyte counts compared WBC (**a**), neutrophil (**b**), lymphocyte (**c**) and T cell (**d**) counts between primary, rechallenge FIPV infections and uninfected SPF cats. Cell counts were determined at weekly time points until euthanasia or the end of the study (week 4 PI). T cell counts were calculated by percentage of CD3+ cells within the total PBMC gate detected by flow cytometry and absolute lymphocyte counts. Symbols represent cell count values for individual cats and dotted lines represent the fitted slope for each cat cohort. Each *P* value represents a comparison of slopes between primary infection and the uninfected control group. Asterisks *** reflect values for *P* < 0.001, ** reflect *P* values < 0.01, and * reflects *P* values < 0.05

**Fig. 2:**
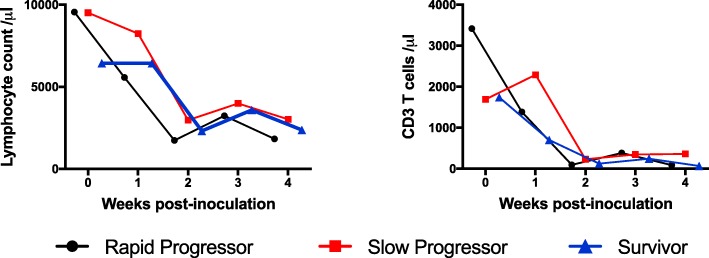
Lymphopenia and T cell depletion associated with different disease outcomes for primary infection. Median values for lymphocyte and T cell counts calculated for rapid progressors, slow progressors, and survivors are plotted for weekly time points of primary infection. Significant differences were not detected for lymphocyte or T cell counts between different disease outcomes at each time point based on analysis by a Kruskal Wallis test

Differential blood cell counts and T cell frequencies were examined longitudinally following primary and secondary infections. Total white blood cell (WBC) counts decreased significantly following primary infection (*P* < 0.001) when compared to healthy age-matched controls (Fig. 1A). The decline in total white cells during acute primary infection was not associated with a decline in neutrophils (Fig. 1B) but rather to a significant decrease in total lymphocyte and T cell counts when compared to uninfected controls, (*P* < 0.001) and (*P* = 0.01) respectively (Fig. 1C-D). However, WBC and absolute lymphocyte and T cell counts did not significantly decline in survivor cats after a second inoculation with FIPV-i3c2 during a rechallenge infection (Fig. 1A, C-D). Interestingly, differences in the magnitude of lymphocyte or T cell depletion were not associated with disease course as survivors showed declines similar to those observed in rapid and slow progressors for both parameters over the four week time period following primary inoculation (Fig. 2). Significant differences in either lymphocyte or T cell counts were not observed between these three animal groups stratified by outcome during acute primary infection based on analysis by a Kruskal Wallis test conducted for weeks 0–4.

### Antiviral antibody responses

Antibody titers ranged from < 1:25 to 1:400 for all cats that developed FIP, except for two cats that survived 49 days and 106 days and demonstrated terminal titers of 1:1600 and 1:6400, respectively (Table 2). Titers ranging from 1:100 to 1:400 at four weeks PI were observed for the four cats that did not develop FIP during primary FIPV infection. Interestingly, all four survivor cats from the acute infection group (11–147, 11–149, 11–166, 11–225) demonstrated a higher anti-FIPV antibody at the time of rechallenge (Table 3), compared to titers observed at week 4 of acute infection for these same cats (Table 1), suggesting persistent viral antigen exposure despite resistance to development of FIP. All survivor cats demonstrated a four-fold increase of antiviral antibody over a four week period after rechallenge with FIPVi3c2 (Table 3).

### Virus detection

Virus detection or virus loads in blood did not correlate with development of FIP based on viral RNA concentrations in either plasma or PBMC for 4/8 (50%) rapid progressors, 3/7 (43%) slow progressors, and 2/4 (50%) survivors (Table 2). Virus was detected most frequently at two weeks PI with 7/9 (78%) viremic cats showing circulating virus in either plasma, PBMC or both, at this time point. In contrast, circulating virus was detected in one cat at one week and two cats at three weeks PI. Circulating virus was also detected in one rapid progressor at a terminal time point bleed (TB). Frequency of virus detection in PBMC (5/9) (56%) of viremic cats was comparable to that observed to plasma (6/9) (67%). However, virus loads tended to be higher in plasma, ranging from 3.1 × 10^4^ to 1.5 × 10^6^ copies per ml of plasma compared to 1.8 × 10^2^ to 2.5 × 10^4^ copies per million PBMC. Viral loads of 3.1 × 10^4^ and 3.2 × 10^5^ copies per ml were detected in plasma from two survivor cats, indicating that viral loads in plasma or PBMC were not associated with disease or resistance. Preparations of MLN were collected at post mortem from 13/15 cats with FIP and all samples tested were positive for viral RNA with delta CT (FIPV CT – GAPDH CT) values ranging from 7.44 (lower loads) to − 4.2 (highest virus load) (data not charted). Virus loads for MLN showed significant variability but no correlation with rapid or slow disease progression. Virus was also detected in MLN (3.7 × 10^4^ copies million cells) and PBMC (3.9 × 10^3^ copies million cells) drawn at the terminal time point from the single cat that developed FIP upon a secondary exposure to FIPV-i3c2. Otherwise, virus was not detected in PBMC or plasma from the nine cats that remained healthy after secondary challenge with FIPV-i3c2.

### Virus-specific T cell proliferation responses in peripheral blood during primary infection

**Fig. 3:**
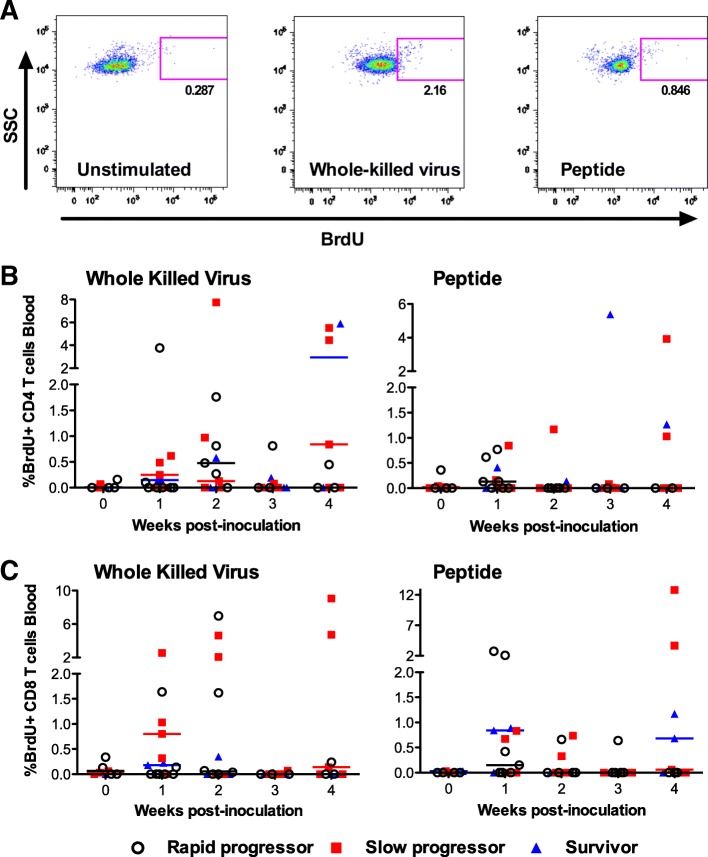
Antiviral T cell proliferative responses during primary FIPV infection. Representative FACS plots comparing unstimulated to WKV and viral peptide stimulation in CD3 + CD4+ T cells are shown (**a**). PBMC were assessed for viability by live/dead exclusion and interrogated by staining for CD3, CD4 or CD8 as described in Methods. T cell subsets were then assayed for proliferation responses to different viral immunogens based on positive staining for BrdU using an anti-BrdU monoclonal antibody. A comparison of the frequencies of CD4 and CD8 cells showing proliferation after stimulation with WKV or FIPV peptide stimulation is shown for rapid progressors, slow progressors and survivors (FIP resistant cats) before and after FIPV inoculation (**b**). Horizontal lines represent median values for each group. Significant differences were not detected for T cell frequencies between different disease outcomes at each time point based on a pair-wise analysis by one-tailed Mann-Whitney U test

T cell proliferation responses following specific antigen stimulation and based on BrdU incorporation were assayed longitudinally and compared for cats with rapid disease, slower progressive FIP, and resistance to FIP. Due to protocol limitations on the blood volumes sampled and infection-associated lymphopenias, antiviral T cell response assays were not consistently available for each cat at every time point. Representative scatter plots for assay of T cell proliferation following antigen stimulation are shown in Fig. 3A. No significant differences for CD4 or CD8 T cell proliferative responses to either viral immunogen were observed at weeks 1–4 after virus inoculation between cats that either succumbed to, or resisted the development of FIP (Fig. 3B-C). Comparisons between cats that died or resisted disease following primary infection were limited by the low number of survivors (*n* = 4) and the number of surviving cats (2–3) assayed at each time point. Similarly, only three rapid progressors were tested at four weeks PI due to deaths of rapid progressors at earlier time points. Despite these limitations, certain trends in antiviral immune responses were demonstrated between the three groups of cats. The emergence of CD8 T cell responses to WKV was detected more frequently in slow progressors (4/5 tested) and survivors (2/3 tested) at one week PI compared to rapid progressors (2/7 tested) (Fig. 3C). By two weeks PI, a higher proportion (4/5) of rapid progressor cats showed relatively strong CD4 T cell responses to WKV whereas 3/5 slow progressors and 1/3 survivors demonstrated detectable CD4 T cell responses at this time point (Fig. 3B). In contrast, detection of CD8 T responses to WKV and CD4 and CD8 T cell responses to viral peptides was infrequent at this time point for all groups. The proportion of cats showing detectable CD4 and CD8 T cell responses to either WKV or peptide by week 3 PI was very low or negligible for all three groups of cats, coinciding with a persistent T cell loss in peripheral blood first observed at week 2 PI. However, by four weeks PI, a recovery of antiviral T cell responses including both CD4 and CD8 T cells, were observed for selected slow progressors and survivors (Fig. 3B-C). CD4 and CD8 T cell responses were very low or negligible for rapid progressors at week 4 of infection with the exception of one cat.

### Virus-specific T cell proliferation responses in peripheral blood during rechallenge infection

**Fig. 4:**
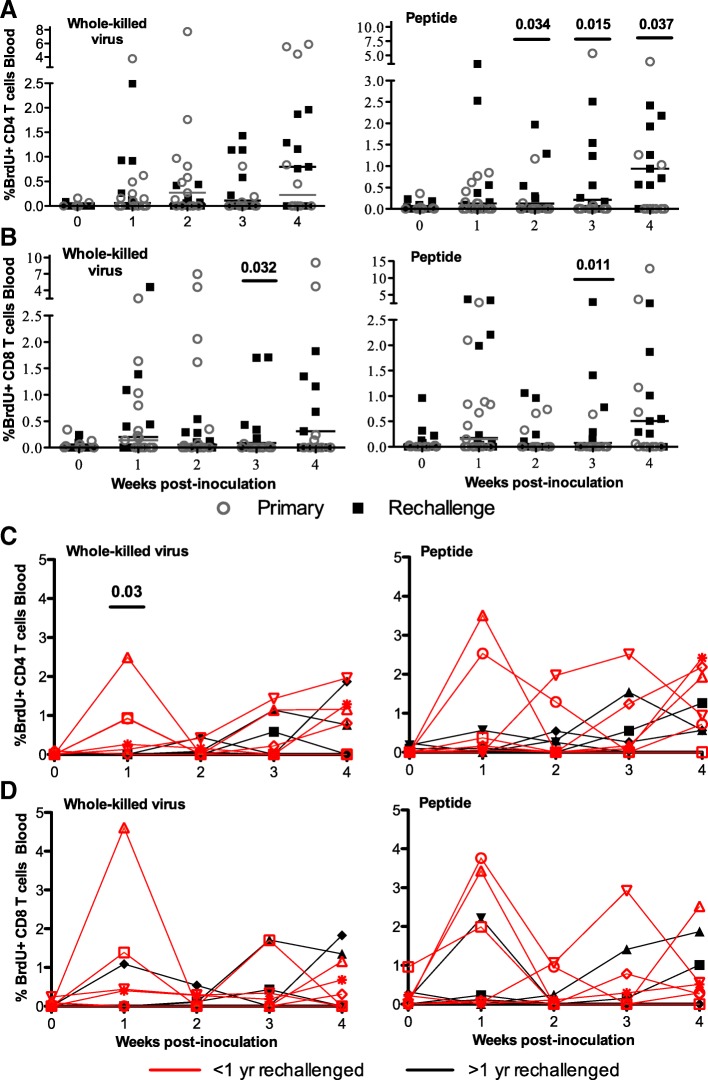
Comparison of antiviral T cell proliferative responses between primary and rechallenge FIPV infections. Frequencies of CD4 and CD8 T cells showing proliferation after stimulation with either WKV or FIPV peptides during primary FIPV infection with cats from all outcomes included, were compared to responses during rechallenge infection (**a**). Horizontal lines represent median values for each group. Frequencies of CD4 and CD8 T cells showing proliferation after stimulation with either viral immunogen were compared between cats rechallenged with FIPV-i3c2 within 6 months of the primary challenge (< 1 yr. rechallenged) and cats that had been inoculated more than 1 year prior to FIPV rechallenge (> 1 yr. rechallenged) (**b**). Numbers shown above any time-point data represent *P* values calculated using a one-tailed Mann-Whitney U test (GraphPad Prism) to compare median T cell proliferation frequencies determined for primary versus rechallenge infections for each time point (**a**) or determined between rechallenge infection groups for each time point of rechallenge infection (**b**). *P* values < 0.05 were considered significant

A cohort of cats surviving previous challenge with FIPV-i3c2 allowed examination of memory antiviral T cell responses induced by re-exposure to the same pathogenic FIPV isolate and the comparison of such responses to those associated with primary infections (Fig. 4A). A small subset of survivor cats revealed detectable baseline CD8 T cell responses to peptides (Fig. 4A). Otherwise, circulating CD4 T cell responses to both peptides and WKV, and CD8 responses to WKV, were low or negligible prior to rechallenge. CD4 T cell proliferation responses to viral peptides were significantly different between primary and rechallenge infections for weeks 2 (*P* = 0.034), 3 (*P* = 0.015) and 4 (*P* = 0.037) where higher CD4 T cell responses were observed for rechallenged survivor cats. CD8 T cell responses to WKV and viral peptides were also higher for rechallenge infections for week 3 (*P* = 0.032 and *P* = 0.011 respectively). As mentioned above, significant declines in circulating lymphocyte and T cell counts were observed during primary infection likely impacting antiviral T cell responses in blood. In contrast, similar declines in lymphocyte and T cell counts were not associated with rechallenge infection. It is noteworthy that cats within the primary infection group showing responses similar to the highest responders in the rechallenge group at the week 4 time point tended to be slow progressor or survivor cats (Fig. 3B-C and Fig. 4A).

Based on the wide range of T cell responses observed for rechallenge infection, this group was further stratified into two cohorts based on timing of the previous exposure to FIPV-i3c2 (Fig. 4B). Cats that were rechallenged with FIPV within 2–6 months after primary infection showed greater CD4 and CD8 T cell responses at one week after rechallenge compared to cats infected one year or more before rechallenge (Fig. 4B). However, this observation was only significant for CD4 T cell responses to WKV (*P* = 0.03). T cell responses were comparable between both groups of cats at weeks 2–4 during rechallenge infection, regardless of time duration between primary and rechallenge infections. Importantly, all cats within the rechallenge infection group demonstrated antiviral T cell responses for multiple time points after a secondary FIPV exposure.

### Virus-specific T cell proliferation responses in blood and lymph nodes associated with FIP

**Fig. 5:**
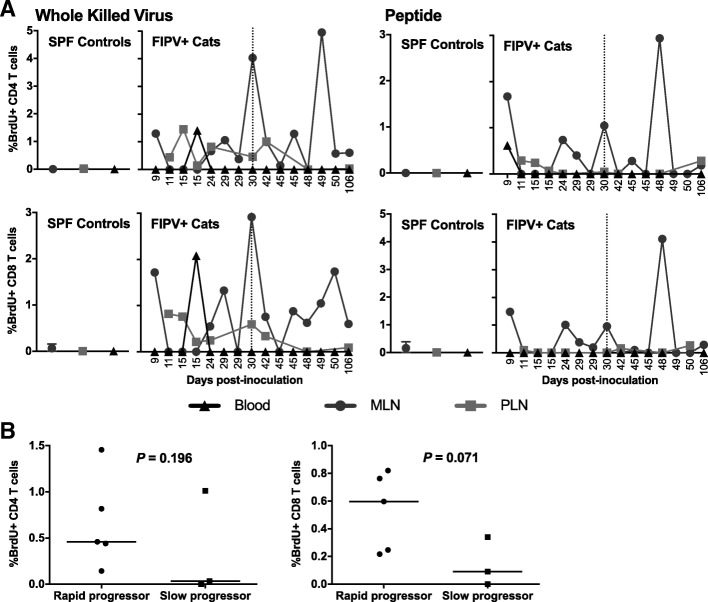
Comparison of antiviral T cell proliferative responses in different lymphoid compartments in cats with FIP. Data plotted for each time-point PI on each graph represent T cell responses (frequencies of proliferating cells) detected for LMNC isolated from either a peripheral lymph node (PLN) or mesenteric lymph node (MLN) and for PBMC (blood) from an individual animal euthanized at that particular time-point due to FIP with terminal time-points representing 14 cats up to 106 days PI. The vertical dotted line for each graph divides time-points of euthanasia for cats showing rapid versus slow progression of disease. Controls represent T cell responses measured in similar lymphoid tissues and blood harvested from healthy uninfected age-matched SPF cats (*n* = 2). Responses for both T cell subsets for two different FIPV immunogens (WKV and viral peptides) are also shown (**a**). Values for T cell responses associated with PLN measured in cats showing rapid progression were compared to those detected for cats showing slower progression of disease and measured at later time points of infection (**b**). Numbers shown for each comparison represent *P* values calculated using a one-tailed Mann-Whitney U test (GraphPad Prism). *P* values < 0.05 were considered significant

The final analysis of this study compared antiviral T cell responses between LNMC harvested from MLN and PLN with PBMC collected at the time of euthanasia due to onset of FIP. PBMC and LNMC were similarly assayed for virus-specific CD4 and CD8 T cell proliferation responses to both viral immunogens, although data for PLN were not available for all cats undergoing post mortem exam. A longitudinal assessment of lymph node T cell responses over time after infection was possible due to temporal differences in the disease course for different cats with FIP. Assay of lymph node T cell responses from two different cats at the same time point of primary infection (15, 29, and 45 days PI) were also available. Responses from both rapid and slow progressors were compared with each other with LNMC harvested from age-matched healthy control cats serving as negative controls. CD4 and CD8 antiviral T cell proliferative responses varied between different tissues within individual cats, between different cats, and between different time points (Fig. 5A). However, CD4 and CD8 T cell responses of the highest magnitude (> 1% proliferating T cells) were observed for MLN (9, 29, 30, 45, 48, 49, and 50 day PI) and blood (15 days PI) when tested against either WKV or viral peptides (Fig. 5A). CD4 and CD8 T cell responses to peptides were usually low or negligible for PBMC, with the exception of PBMC harvested at 9 and 15 days PI from two rapid progressor cats. Overall, higher MLN CD4 and CD8 T cell responses to WKV were observed in cats that developed FIP after 24 days of primary infections suggesting an association of more robust T cell responses at this particular site with longer survival, although differences between rapid and slow progressors were not statistically significant. Also of importance was a frequent lack of a comparable magnitude in antiviral T cell responses between PLN and MLN from the same cat suggesting that a PLN may not serve as a surrogate site for assay of host MLN responses. However, a trend for PLN CD8 T cell responses to WKV of greater magnitude was observed for rapid progressors (*n* = 5) at terminal time points compared to PLN responses observed for slow progressors (*n* = 3) (Fig. 5B), although the difference was not statistically significant (*P* = 0.071).

## Discussion

Although multiple reports have described immune responses associated with FIPV infection, immune correlates that protect infected animals from this fatal disease remain poorly understood. Previous studies investigating antiviral T cell responses induced by experimental infection with either FIPV79–1146 or FIPV KU-2, were limited to a single time-point after infection and provided evidence supporting the role of T cell immunity in resistance to FIP [6–8]. This report describes the first longitudinal study of virus-specific T cell responses in cats experimentally infected with a highly pathogenic type 1 FIPV isolate (FIPV-i3c2). The aim of this study was to identify possible antiviral T cell correlates for disease outcome during acute infection and after rechallenge of FIP-resistant cats with the same FIPV isolate. Two approaches were used to characterize such correlates including a comparison of T cell immune responses in cats that either exhibited a rapid or slow progression to FIP, or did not develop FIP during primary infection with FIPV-i3c2. A second approach involved comparison of T cell responses in cats during primary FIPV-i3c2 infection with responses elicited after a rechallenge exposure of cats that survived a primary FIPV challenge. A reasonable hypothesis for host factors responsible for absence of FIP would be the emergence of circulating antiviral T cell responses of significantly higher magnitude during the first two weeks of infection. However, our findings did not reveal a definitive correlation for emerging antiviral T cell responses during very early stages of primary infection and disease outcome. Nevertheless, a recovery of antiviral T cell responses at a later time point of primary infection and emergence of antiviral T cell responses in FIP-resistant cats after a second exposure to FIPV suggested that cellular immunity may be associated with some level of control of FIPV infection resulting in a delay or absence of disease progression.

Both WKV and S2 peptides proved capable of evoking CD4 and CD8 T cell proliferative responses in cats experimentally infected with FIPV-i3c2, and implemented a valuable tool for a longitudinal investigation of antiviral T cell responses during primary and rechallenge infections. Limitations to these studies included the low number of survivor cats during primary infection and assessment of T cell responses restricted to blood only, for early time points of primary infection. In contrast, lymph node and circulating T cell responses were compared at terminal time points in cats that progressed to disease. A comparison of responses in abdominal lymphoid tissues at earlier time points of infection for both progressors and survivors would be more informative based on the typical localization of FIPV replication to the abdominal cavity. Despite these limitations, examination of antiviral T cell responses in blood during primary infection revealed certain trends and critical observations. An earlier emergence of both CD4 and CD8 T cell responses observed for slow progressors and survivors at one week after virus inoculation contrasted to a delay of one week in responses emerging in some cats with rapidly progressive disease. Regardless of these temporal differences in early responses, the magnitude of T cell responses did not differ significantly between cats with different disease outcomes during the first three weeks of early infection and almost all cats showed negligible responses by three weeks into infection. However, by four weeks after infection, a proportion of slow progressors and survivors were distinguished from rapid progressors in their recovery of antiviral T cells responses. Collectively, these findings suggested that circulating antiviral T cell responses during very early time points of acute infection do not predict disease outcome. Yet, recovery of circulating antiviral T cell responses later in acute infection may be associated with a delay in disease or absence of disease. These findings lend some support to the widely-held hypothesis that FIP results from a failure of T cell immunity [1], but also suggest that other undetermined virus host interactions or antiviral responses are critical for an outcome of FIP.

Robust CD4 and CD8 T cell responses were more frequently demonstrated in MLN harvested from more cats surviving 29 days or later. In contrast, T cell responses in PLN harvested at earlier time points of primary infection tended to be of higher magnitude compared to PLN responses observed for slow progressors. Also, PLN-associated T cell responses frequently did not match MLN responses from the same host, particularly for cats surviving 29 days or later*.* These findings suggest that MLN may represent a major site for T cell-mediated control of virus replication and disease progression and FIPV responses in both disease and antiviral immunity. This finding may not be surprising based on previous reports showing virus replication in systemic lymphoid tissues including MLN [28–30] and a relative absence of detectable virus in PLN [31]. Furthermore, previous reports revealed that the mildest form of FIP disease was limited to the MLN, and that some cats with this form of disease will proceed to recovery further suggesting a role for antiviral immunity at this tissue site. Conversely, dissemination of virus infection from MLN to other sites within the host is associated with disease progression [1, 32].

The most striking abnormalities observed during primary FIPV infection were a progressive decline in WBC, lymphopenia, and T cell depletion, which most likely impacted host responses to the virus infection during the early phases of acute primary infection. This is not a new observation and was described in other reports of natural and experimental FIPV infections in cats developing FIP [6, 31, 33, 34]. An unexpected finding was the observation of similar profound changes in the T cell compartment in cats that survived the infection with no outward signs of disease during primary infection. A comparable finding of T cell depletion was also reported for cats that recovered without FIP after infection with pathogenic type 2 FIPV 79–1146 [6]. However, findings also reported by our group for one survivor and one rapid progression for FIPV-m3c2 from a different study revealed lymphopenia demonstrated only by the rapid progressor [30]. Whether differences specific to the FIPV isolate, route of inoculation (intraperitoneal inoculation for FIPV-m3c2 study) or other unknown variables accounted for the lymphopenia and T cell depletion observed in this study for survivors remains to be determined. The T cell depletion observed for all FIPV-i3c2-infected cats regardless of subsequent disease progression provides one compelling explanation for the demise of detectable circulating antiviral T cell responses by three weeks into primary infection for all cats and a failure to characterize cellular immune responses associated with survival.

A recent experimental FIPV infection study by our group revealed that sustainable immunity is not always maintained upon multiple exposures of the virus suggesting a distinct antiviral T cell response may confer resistance to FIP among survivor cats [2]. Rechallenge of survivor cats with the same pathogenic FIPV as described in this report, offered an important opportunity to examine the magnitude of T cell responses after a secondary exposure to FIP and to also compare such responses to those associated with primary infection. Moreover, this rechallenge study determined whether survivor cats would be capable of maintaining resistance to a second challenge with pathogenic FIPV-i3c2. Results revealed that nine out of ten cats surviving a primary infection remained resistant to FIP after a secondary exposure to infectious FIPVi3c2, regardless of the span of time between primary and second challenge with FIPV. In contrast, only one survivor cat subsequently developed FIP. Antiviral T cell responses noted after FIPV rechallenge were variable in magnitude, particularly at one week PI where variability was associated with the duration of time between the primary and secondary infections. Irrespective of variability between cats, all rechallenge infections were associated with reappearance of antiviral T cell responses with detection of T cell responses observed for 2–3 time points after rechallenge. In addition, rechallenge infection showed greater antiviral T cell responses for multiple time points after FIPV rechallenge including week three, compared to primary infection responses. This finding is noteworthy because the week three time point in primary infection was characterized with ablated antiviral T cell responses and significant T cell depletion for all cats including slow progressors and survivors. Furthermore, cats that survived an earlier infection with FIPV not only failed to develop clinical symptoms of FIP but also demonstrated very mild or no lymphocyte and T cell depletion after rechallenge with pathogenic FIPV-i3c2. The absence of severe lymphoid depletion and other clinical signs of FIP in survivor cats during rechallenge infection, along with detection of antiviral T cell responses over multiple time points including three weeks PI, implies a role for cellular immunity in the resistance to FIP after a secondary exposure to the virus. It should be noted that the survivor cat that developed FIP after rechallenge demonstrated antiviral T cells responses during early time points post rechallenge but responses were absent by 3 weeks post rechallenge, the final time point for this cat. Persistence of antiviral T cell responses has been demonstrated in human patients recovered from the Severe Acute Respiratory Syndrome (SARS) coronavirus (SARS-CoV) [35, 36]. However, antiviral humoral responses including neutralizing antibody, as well as other types of host responses must also be considered as possible factors for this resistance to disease after a secondary exposure to FIPV.

Viremia was not an accurate predictor of disease outcome and was observed in either plasma or PBMC in approximately 50% of cats that either died or survived primary infection, with circulating virus most frequently observed at two weeks PI. This time point coincides with the emerging lymphocyte and T cell depletion and compares well to results of experimental studies with pathogenic Type 2 FIPV 79–1146 where viremia was typically restricted to 4–12 days PI, when detected [6, 8]. Viral RNA was consistently detected in mesenteric lymph nodes at post mortem for cats with FIP, which confirmed this tissue as a consistent site for virus replication. This is in agreement with findings in another study measuring the levels of FIPV in various tissues where high levels of virus were detected in diseased tissues particularly the omentum and MLN, but not in blood [30]. In contrast, survivor cats did not develop a detectable viremia after rechallenge with FIPV-i3C2, with the exception of the one cat that developed FIP after a secondary exposure and tested positive for viral RNA in blood and mesenteric lymph node at post mortem. Increases by four-fold in antiviral antibody titers and emergence of memory T cell responses observed for all survivor cats during the four weeks after rechallenge with FIPV-i3c2 suggested the possibility of some level of virus replication resulting from the second exposure that was nevertheless controlled by host responses. Questions regarding sites of persistent virus replication or virus elimination for cats apparently resistant to FIP after FIPV-i3c2 infection, warrant further investigation including assessment of multiple tissues for viral RNA as described in companion pathogenesis studies of this virus isolate by N.C. Pedersen et al. [30].

Taken together, findings from these infection studies did not reveal definitive immune correlates for resistance, or a delay in progression to FIP during acute primary infection with a pathogenic type 1 FIPV, except for a recovery of antiviral T cell responses at a later time point of infection for some but not all of the slow progressors and survivors tested. Intriguing findings were that antiviral T cell responses, circulating virus loads, or the magnitude of T cell depletion immediately after a primary exposure to pathogenic FIPV, did not appear to predict disease resistance in this experimental cohort. However, those cats that recovered FIPV-specific T cells responses without developing FIP during a primary infection, proved capable of resisting a second challenge with FIPV that was associated with emergence of recall T cell responses.

Critical virus-host interactions very early in infection, which are responsible for resistance or slow progression to disease, remain unclear from our experimental primary infection studies. Possible explanations include differences in the magnitude of disruption of innate responses or specific alterations of innate factors that have been reported for both SARS CoV and FIPV. Virus-induced inhibitory macrophages reported for the mouse model for the SARS-CoV [37] were shown to contribute to reduced dendritic cell activation with subsequent delay of virus-specific T cell responses and disease onset. Other inappropriate innate responses included unregulated proinflammatory cytokine responses leading either to a cytokine storm as reported for the SARS CoV [38, 39] or to an overstimulation of B cells [40] possibly leading to enhancing antibody responses as described for the classical wet form of FIP [1, 41, 42]. Similar to SARS, overexpression of inflammatory cytokines including IL-6 and tumor necrosis factor (TNF)-α with a reduced expression of IL-10 have also been reported to be associated with the development of FIP [42]. Production of proinflammatory cytokine TNF-α from FIPV-infected macrophages has also been implicated in FIPV-associated lymphoenia [43, 44]. Moreover, a recent report revealed that targeting TNF-α with an anti-feline TNF-α monoclonal antibody prevented infection in cats experimentally infected with FIPV [45]. Pro-inflammatory cytokines and antiviral-related genes such as MX1, viperin and IFNγ have also been observed in tissues harvested from FCoV-infected cats with FIP [46]. In addition, recent reports focused on gene expression profiles from mesenteric or peritoneal macrophages harvested from cats with FIP revealed expression of pattern recognition receptors including toll, NOD and RIG-like receptors, pro-apoptotic genes, and genes related to differentiation of M1 macrophages in contrast to reduced expression of MCH class II receptor genes [47, 48]. Lastly, the role of host anti-inflammatory factors such as regulatory T cells and IL-10 as well as innate factors such as natural killer cells factors warrant further examination based on other recent reports [29, 49]. Future studies will need to simultaneously address both innate and adaptive responses within critical tissue sites during very early stages of infection to answer these critical questions regarding mechanisms of recovery from acute pathogenic FIPV infection.

## Conclusions

Key findings from these studies indicate that circulating antiviral T cell responses within the first four weeks of primary infection with pathogenic FIPV isolate, were not predictive of disease outcome. Furthermore, all cats developed lymphopenia and T cell depletion during early FIPV infection regardless of progression or resistance to FIP. Findings that suggest a role for cellular immunity in FIPV pathogenesis were a recovery of antiviral T cell responses at a later time point of primary infection for a subset of cats showing slow progression or resistance to disease. T cell responses measured in MLN harvested post mortem were more robust and more frequent for cats showing slower progression to FIP. Lastly, secondary or recall antiviral T cell responses observed in FIP-resistant cats after a second exposure to FIPV were associated with resistance to disease progression as well as an absence of lymphopenia and T cell depletion. Overall these findings suggest that antiviral T cell immunity may contribute to FIPV pathogenesis and resistance to disease, but also provoke questions regarding the role of other host factors including innate response factors, immunoregulatory responses, and B cell alterations that may also determine disease outcome.

## Abbreviations

AF: Alexa fluor
APC: Allophycocyanin
BrdU: Bromodeoxyuridine
CBC: Complete blood cell count
CCR: Chemokine receptor
DMSO: Dimethyl sulfoxide
fcwf-4: *Felis catus* whole fetus-4
FECV: Feline enteric coronavirus
FIP: Feline infectious peritonitis
FIPV: FIP virus
FITC: Fluorescein isothiocyanate
GAPDH: Glyceraldehyde 3-phosphate dehydrogenase
HR: Heptad region
IFA: Indirect immunofluorescence antibody
IFN-γ: Interferon gamma
IH: Inter-helical
IL: Interleukin
LNMC: Lymph node mononuclear cells
MLN: Mesenteric lymph node
PBMC: Peripheral blood mononuclear cells
PE: Phycoerythrin
PEG: Polyethylene glycol
PI: Post infection
PLN: Peripheral lymph node
S2: Spike 2
SPF: Specific pathogen free
TB: Terminal time point bleed
UCD: UC Davis
WBC: White blood cell
WKV: Whole-killed virus
